# Recurrent Aphasic Seizures as the Initial Manifestation of LGI1 Antibody-Associated Autoimmune Limbic Encephalitis Mimicking a Transient Ischaemic Attack: A Case Report

**DOI:** 10.7759/cureus.116958

**Published:** 2026-09-26

**Authors:** Aima Hameed, Jyotsna Kakarla, Siddhi A Gaunekar, Akshayaa K Aggarawal, Ajmal Raoof

**Affiliations:** 1 General Internal Medicine, Kettering General Hospital NHS Foundation Trust, Kettering, GBR; 2 General Surgery, Queen's Hospital Burton, University Hospitals of Derby and Burton NHS Foundation Trust, Burton-on-Trent, GBR; 3 Internal Medicine, Walsall Manor Hospital, Walsall, GBR; 4 Internal Medicine, Kettering General Hospital NHS Foundation Trust, Kettering, GBR

**Keywords:** autoimmune limbic encephalitis, corticosteroids, epileptic seizures, focal seizures, ischaemic cerebrovascular disease, motor aphasia

## Abstract

We report the case of a 55-year-old man who presented with recurrent, brief episodes of isolated speech arrest lasting 10-30 seconds, during which awareness and comprehension were preserved. Initial neurological examination and neuroimaging were unremarkable, and the episodes were initially attributed to suspected transient ischaemic attacks (TIAs).

Subsequently, the patient developed a generalised tonic-clonic seizure, prompting initiation of antiepileptic therapy. Over the following months, he experienced an increasing frequency of transient aphasic episodes alongside focal seizures with left-sided motor involvement, which proved refractory to multiple antiepileptic medications.

Further investigation during hospital admission revealed significant hyponatraemia and characteristic magnetic resonance imaging (MRI) findings, including bilateral medial temporal lobe enlargement with fluid-attenuated inversion recovery (FLAIR) hyperintensity involving the amygdala and hippocampi. The detection of LGI1 antibodies, in conjunction with the clinical presentation and radiological features, confirmed the diagnosis of LGI1 antibody-associated autoimmune limbic encephalitis.

The patient was treated with intravenous methylprednisolone followed by oral corticosteroid therapy, resulting in a marked reduction in seizure frequency and severity. This case underscores the importance of recognising aphasic seizures as a potential manifestation of autoimmune encephalitis (AIE), particularly LGI1-associated disease, to avoid diagnostic delay and facilitate early initiation of immunotherapy.

## Introduction

Autoimmune encephalitis (AIE) comprises a heterogeneous group of immune-mediated inflammatory disorders of the central nervous system (CNS), characterised by acute to subacute progressive brain inflammation. Among its various subtypes, anti-N-methyl-D-aspartate receptor (anti-NMDAR) encephalitis is the most frequently identified. AIE is now recognised as one of the leading causes of non-infectious encephalitis and should be considered an important differential diagnosis in patients presenting with unexplained focal neurological deficits [1].

Leucine-rich glioma-inactivated protein 1 (LGI1) antibody-associated encephalitis represents the most common form of autoimmune limbic encephalitis after anti-NMDAR encephalitis [2]. The disease predominantly affects individuals older than 50 years and demonstrates a marked male predominance [3]. LGI1 is a neuronal protein highly expressed within the hippocampus and temporal cortex. Secreted into the synaptic cleft, it interacts with ADAM22 and ADAM23 to form a trans-synaptic complex that stabilises postsynaptic α-amino-3-hydroxy-5-methyl-4-isoxazolepropionic acid (AMPA) receptors, supports hippocampal circuit maturation, and regulates synaptic transmission. Disruption of this interaction by pathogenic LGI1 antibodies results in reduced AMPA receptor density and impaired synaptic signalling. Experimental models have demonstrated decreased AMPA receptor-mediated transmission and increased seizure susceptibility in the setting of LGI1 deficiency, supporting this proposed pathophysiological mechanism [4].

Clinically, LGI1 encephalitis is characterised by a constellation of symptoms, including cognitive impairment, particularly short-term memory deficits, limbic encephalitis, temporal lobe epilepsy, psychiatric manifestations, and hyponatraemia. Faciobrachial dystonic seizures (FBDS), a highly characteristic feature of the disease, occur in approximately one-third to one-half of affected patients and frequently precede the development of other neurological manifestations [5]. Biochemically, hyponatraemia is a nonspecific finding; it is reported in up to 60% of cases and serves as an important supportive clinical clue [6].

Electroencephalography (EEG) has emerged as a valuable diagnostic tool in the evaluation of suspected AIE. EEG findings commonly include temporal slowing, focal epileptiform discharges, and focal seizure activity. In anti-LGI1 encephalitis, EEG abnormalities are frequently observed, with epileptiform discharges representing the most common finding, followed by focal slowing predominantly involving the temporal regions [7]. In contrast, magnetic resonance imaging (MRI) and cerebrospinal fluid (CSF) analyses may be normal during the early stages of the disease, making EEG a relatively sensitive modality for initial evaluation. Beyond its utility in seizure detection, EEG also assists in excluding alternative diagnoses, particularly primary psychiatric disorders [8].

Aphasic seizures are a subtype of focal seizure characterised by transient expressive or receptive language dysfunction with preserved awareness [9]. These episodes may present as isolated speech disturbances, frequently mimicking transient ischaemic attacks (TIAs) or other cerebrovascular events, thereby contributing to diagnostic delay and misdiagnosis [10]. Although seizures are a common manifestation of LGI1 encephalitis, aphasic seizures are an uncommon and often underrecognized presentation. Their nonspecific clinical appearance can obscure the underlying autoimmune aetiology, particularly in the absence of more characteristic features such as FBDS or overt cognitive impairment.

The diagnosis of LGI1 encephalitis during its early stages remains challenging because of its heterogeneous and evolving clinical presentation. Symptoms typically develop in a subacute fashion, and initial investigations may be unrevealing. Neuroimaging, EEG, and CSF studies can be normal or demonstrate only subtle, nonspecific abnormalities, further complicating diagnostic evaluation. As a result, a high index of clinical suspicion is required, especially in patients presenting with unexplained recurrent focal seizures or transient neurological deficits. In such cases, serial neuroimaging, prolonged EEG monitoring, and serum or CSF antibody testing are often necessary to establish the diagnosis and facilitate timely initiation of immunotherapy [11].

Prompt recognition of LGI1 encephalitis is essential, as the condition is highly responsive to immunotherapy. Corticosteroids alone or combined with other agents (intravenous IG or plasmapheresis) are given as first-line therapy [12]. Early treatment has been associated with improved seizure control, reduced disease burden, and better long-term cognitive outcomes [13]. In this report, we present a patient who presented with recurrent aphasic seizures as the initial manifestation of LGI1 antibody-associated autoimmune limbic encephalitis.

## Case presentation

A 55-year-old man presented to the Emergency Department with a transient episode of inability to speak lasting approximately 20-30 seconds. To our knowledge, this was the first documented presentation of such an event. The episode occurred while he was seated and reading. During the episode, awareness was preserved, and he was reportedly able to understand spoken commands, as described by both the patient and his wife. Formal language testing was not performed during the brief event, but the history was most consistent with speech arrest or expressive aphasic seizure rather than receptive aphasia. There was no associated motor activity, weakness, sensory disturbance, or loss of consciousness. The symptoms resolved spontaneously before assessment.

His past medical history included hypertension, atrial fibrillation, Wolff-Parkinson-White syndrome status post ablation, benign prostatic hyperplasia, and hay fever. He was in sinus rhythm at presentation, had a CHA₂DS₂-VASc score of 1, and was not anticoagulated. On initial assessment, speech, cognition, and higher cortical functions were intact, and neurological examination was unremarkable.

Following admission, the patient experienced multiple similar brief episodes of transient speech arrest. Initial neuroimaging, including computed tomography (CT) of the head, was normal (Figure 1). Given the transient isolated language disturbance and vascular risk factors, he was referred from the Emergency Department to the TIA clinic.

**Figure 1:**
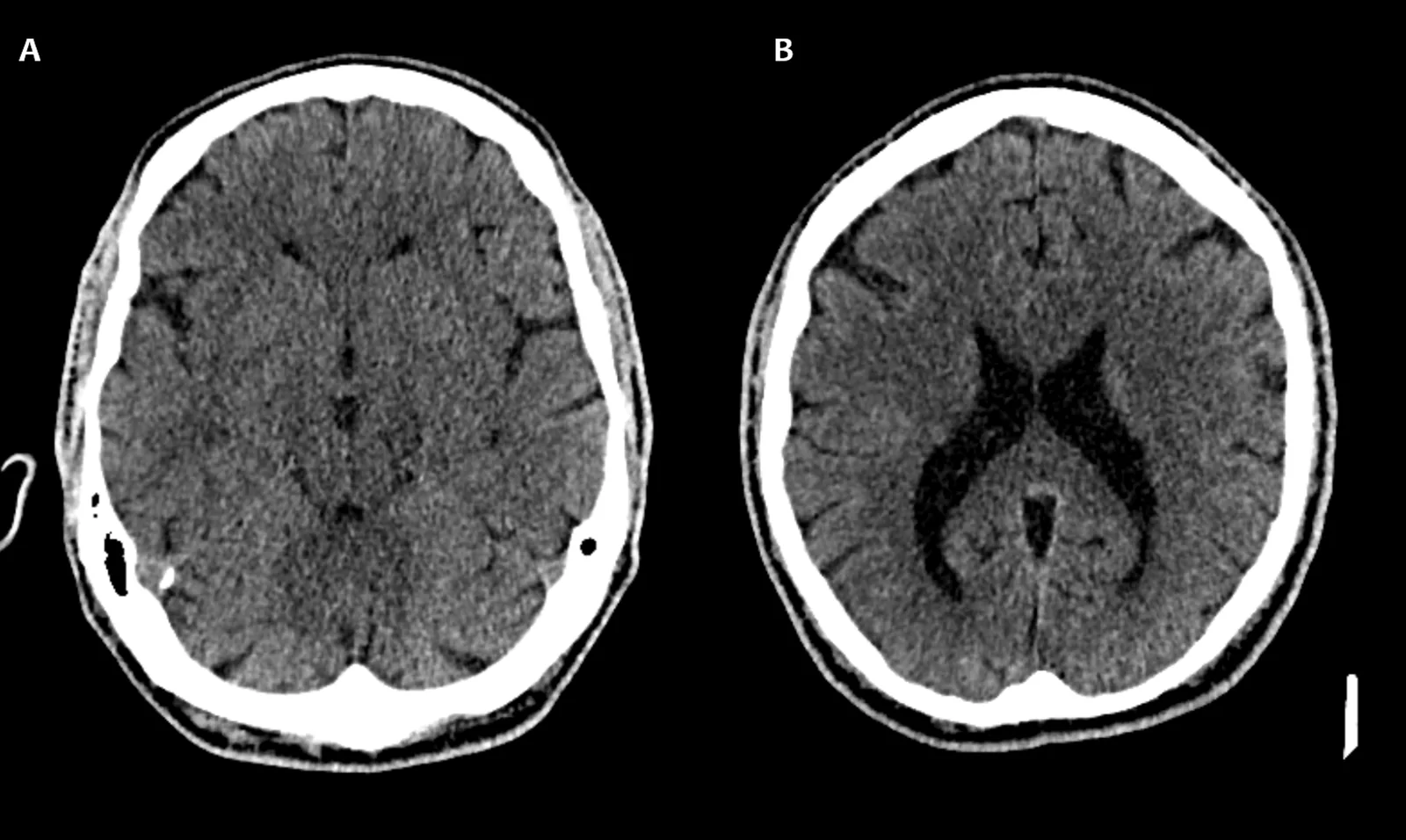
CT head showing no acute pathology Non-contrast axial CT images of the brain (A) and (B) show no evidence of acute intracranial haemorrhage or large territorial infarction. CT: computed tomography

MRI of the brain was performed to rule out cerebral infarction and demonstrated no acute abnormalities (Figure 2). He was discharged with safety-netting advice and secondary stroke prevention therapy, including aspirin 300 mg once daily and atorvastatin 80 mg once daily for 14 days, followed by clopidogrel 75 mg once daily, according to the stroke treatment guidelines.

**Figure 2:**
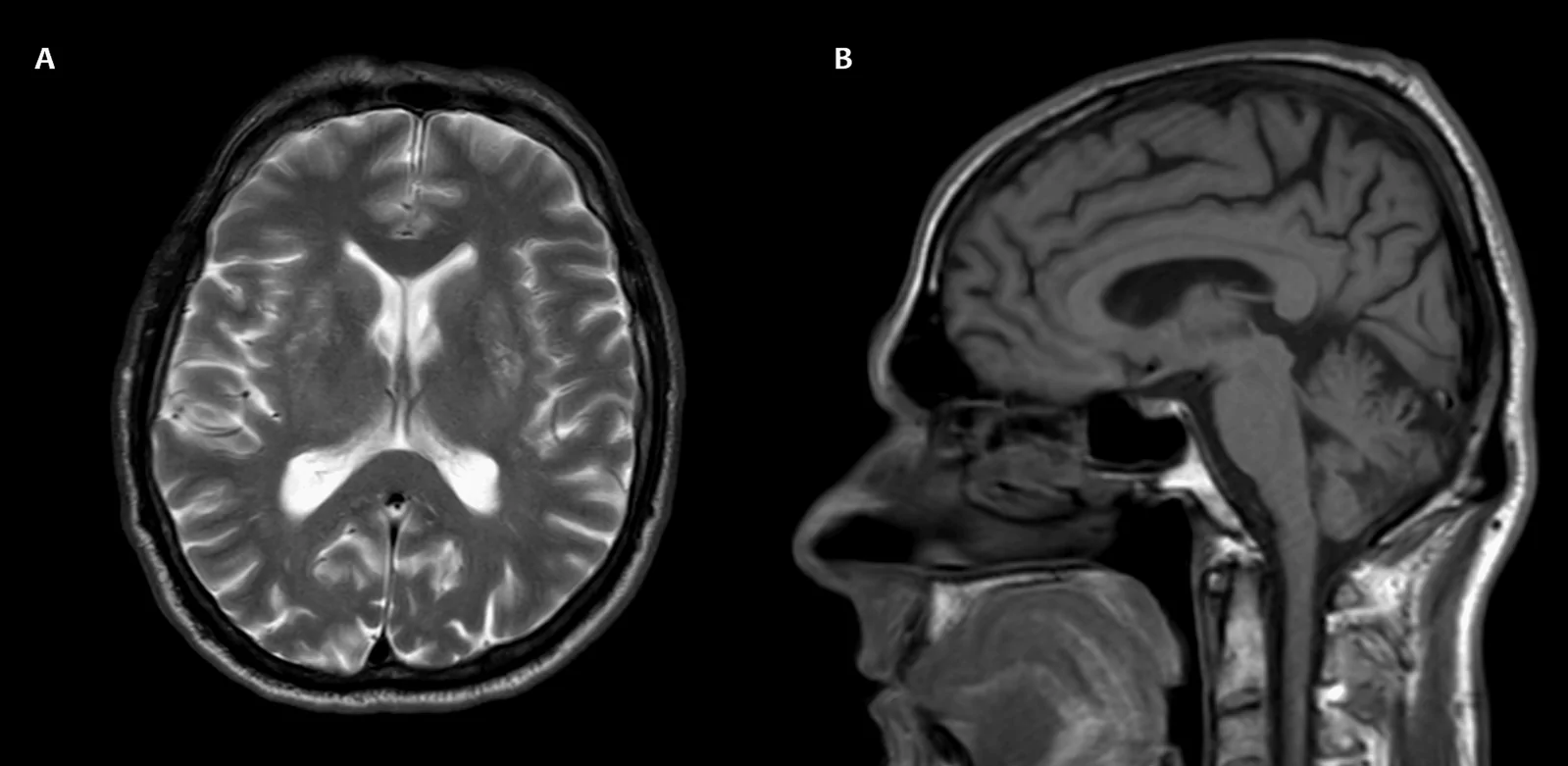
MRI head showing no acute pathology The brain MRI (A) and (B) show no acute pathology or lacunar infarct. MRI: magnetic resonance imaging

At TIA clinic review, the episode was documented as transient with full recovery and was considered more likely to represent seizure activity than TIA. Documentation regarding the rationale for antiplatelet switching and whether anticoagulation was specifically considered in the context of atrial fibrillation was not available in the records. CT angiography or perfusion imaging was not performed, as stroke and TIA were ruled out.

A few days later, he re-presented to the Emergency Department after a five-minute generalised tonic-clonic seizure, followed by a post-ictal state characterised by confusion, agitation, and reduced responsiveness lasting approximately 30 minutes. Neurological examination remained normal after recovery, and a clinical diagnosis of epilepsy was made. He was commenced on levetiracetam 500 mg twice daily, with seizure clinic follow-up arranged.

A 24-hour ambulatory EEG performed in view of seizures showed occasional rhythmic theta activity (5-8 Hz, <60 µV) over the left fronto-centro-temporal region (Figure 3). In response, levetiracetam was gradually escalated to 1500 mg twice daily. The patient was subsequently followed up in the seizure clinic for the management of seizures.

**Figure 3:**
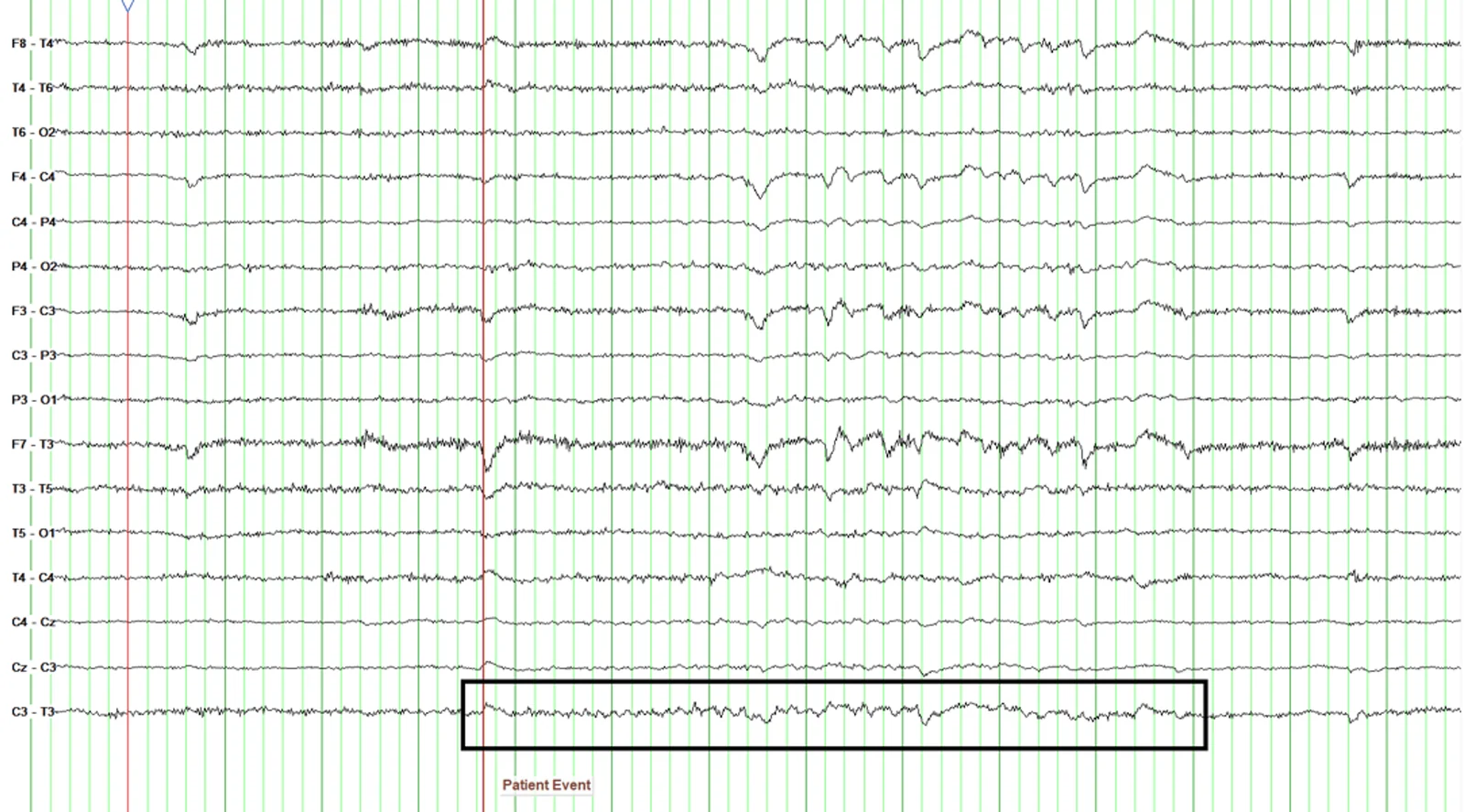
EEG done to investigate seizures The EEG shows a brief change (highlighted over the left centro-temporal channels, where the activity is maximal) when the patient pressed the event marker to indicate an attack. EEG: electroencephalography

Over the following months, despite treatment, the patient continued to experience two distinct seizure types. The first consisted of frequent transient aphasic episodes without impaired awareness, initially occurring 10-20 times daily and increasing to up to 50 episodes per day, each lasting 10-30 seconds with spontaneous resolution. The second seizure type involved brief focal motor events characterised by fidgeting movements of the fingers of the left hand, with associated left-sided facial twitching, without loss of awareness, postural tone, or post-ictal confusion. Due to persistent symptoms, lamotrigine 25 mg once daily was introduced as adjunctive therapy.

In the subsequent months, he re-presented following further generalised tonic-clonic seizure activity witnessed by his wife. There had been no reported non-adherence to his antiseizure medication. Laboratory investigations demonstrated marked hypotonic hyponatraemia, with a serum sodium level of 115 mmol/L. The biochemical profile, together with clinical euvolemia and normal thyroid, adrenal, and renal function, supported a syndrome of inappropriate antidiuretic hormone secretion (SIADH) (Table 1). Other causes of hypotonic hyponatraemia, including hypothyroidism, adrenal insufficiency, renal impairment, and gastrointestinal losses, were not supported by the available clinical and biochemical findings. Reset osmostat was considered less likely given the severity of hyponatraemia and subsequent response to treatment. Lamotrigine was initially considered a potential contributing factor and was therefore discontinued. He was loaded with intravenous lacosamide 200 mg, which was subsequently continued and up-titrated. He was then started on lacosamide 100 mg twice daily for the control of seizures. 

**Table 1:** Work up for hyponatraemia - a possible cause of seizures TSH: thyroid-stimulating hormone

Test	Report	Normal range
Serum sodium	115 mmol/L	133-146 mmol/L
Serum potassium	4.5 mmol/L	3.5-5.3 mmol/L
Serum creatinine	60 µmol/L	59-104 µmol/L
Serum urea	5 mmol/L	2.1-7.1 mmol/L
Spot urine sodium	40 mmol/L	20-40 mmol/L (random)
Spot urine potassium	27.2 mmol/L	25-125 mmol/L (random)
Urine creatinine	8.6 mmol/L	2-20 mmol/L (random)
Serum osmolality	270 mOsmol/kg	275 -295 mOsmol/kg
Urine osmolality	270 mOsmol/kg	50-1200 mOsmol/kg
Cortisol	360 nmol/L	140-690 nmol/L (morning)
TSH	1.5 mIU/L	0.4-4.0 mIU/L

Serum sodium improved following fluid restriction and medication adjustment; however, the frequent stereotyped episodes persisted despite biochemical improvement. This suggested that hyponatraemia alone was unlikely to account fully for the ongoing seizure burden and supported further investigation for an underlying encephalitic process. His wife also reported intermittent confusion, memory difficulties, and fluctuating communication, with periods during which he appeared close to his usual baseline.

CT of the thorax, abdomen, and pelvis, as well as testicular ultrasound, did not reveal any underlying malignancy. These investigations were performed as part of the evaluation for a potential paraneoplastic or malignancy-associated process and to investigate possible causes of SIADH (Figure 4).

**Figure 4:**
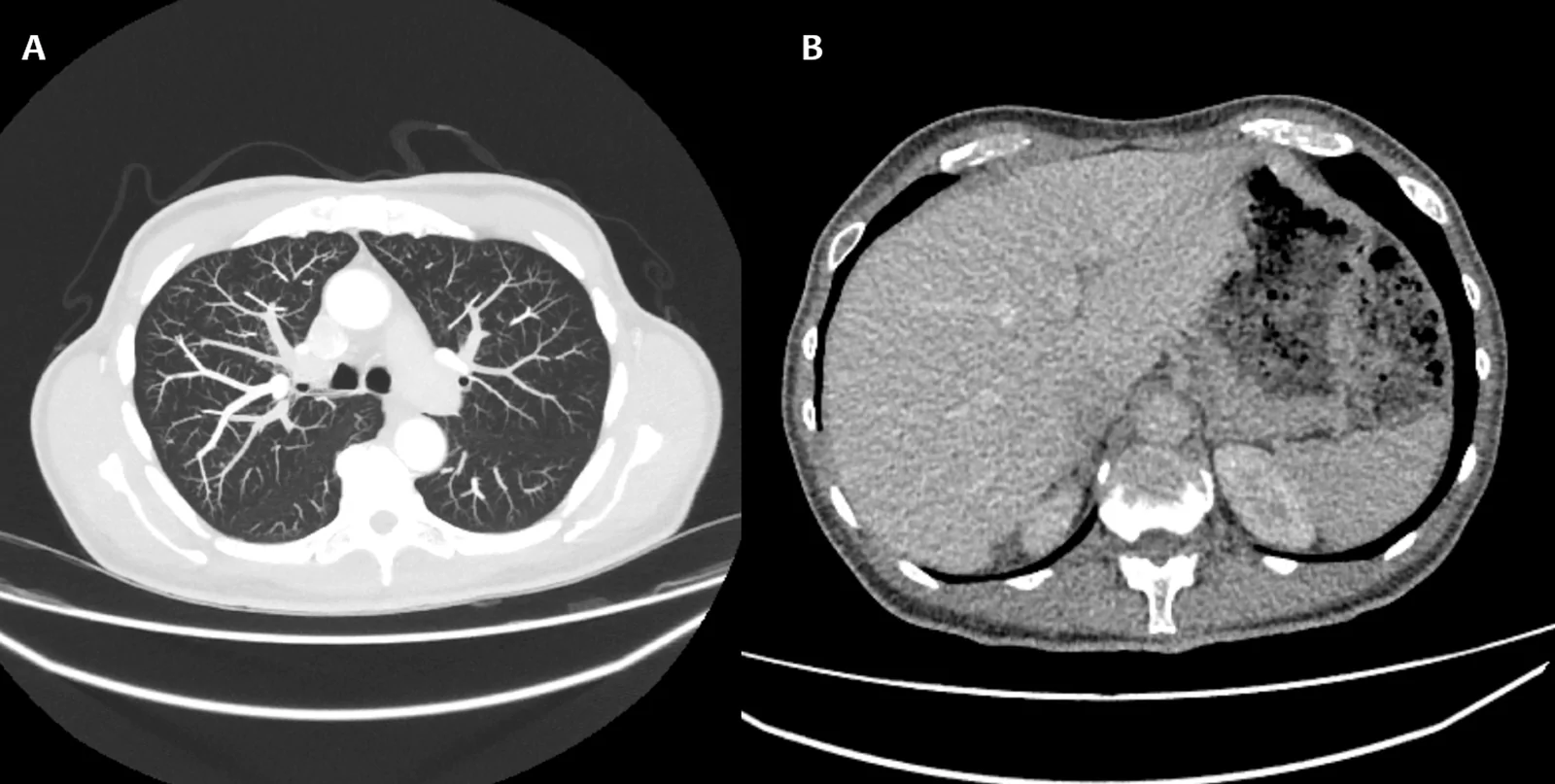
CT of the chest (A) and abdomen (B) showing normal lung parenchyma and no organomegaly No signs of malignancy or abnormal enlargement are seen. CT: computed tomography

During neurological assessment, three brief episodes involving the face and upper limb were directly observed and recognised as FBDS. The combination of highly frequent stereotyped episodes, fluctuating cognitive symptoms, and SIADH-related hyponatraemia raised concern for AIE rather than poorly controlled epilepsy alone.

To investigate the seizures, the patient underwent a lumbar puncture, and CSF analysis was performed. It revealed clear, colourless fluid with normal glucose and protein levels, no pleocytosis, and negative microbiological studies (Table 2).

**Table 2:** CSF analysis CSF: cerebrospinal fluid

Test	Value	Normal value
CSF glucose	3.9 mmol/L	2.7-4.4 mmol/L
Oligoclonal banding	Not present	Not present
Appearance	Colourless	Clear, colourless
White blood cells	1 × 10⁶/L	<5 cells/µL
Red blood cells	6 × 10⁶/L	0 cells/µL
Organisms	No organisms seen	None seen
Culture	No growth	No growth
Aerobic bottle	No growth after 5 days of incubation	No growth
Anaerobic bottle	No growth after 5 days of incubation	No growth

Comprehensive infectious screening using polymerase chain reaction (PCR) detected no DNA or RNA from common pathogens known to cause meningitis or encephalitis, including herpesviruses, enteroviruses, and various bacteria (Table 3). Herpes simplex virus types 1 and 2 PCR were negative. This effectively ruled out infectious causes of the patient’s symptoms. 

**Table 3:** CSF viral screen test CSF: cerebrospinal fluid; RT-PCR: reverse transcription polymerase chain reaction

CSF Test	Result
*Escherichia coli* K1 DNA (BioFire)	Not detected
*Haemophilus influenzae* DNA (BioFire)	Not detected
*Listeria monocytogenes* DNA (BioFire)	Not detected
*Neisseria meningitidis* DNA (BioFire)	Not detected
*Streptococcus agalactiae* DNA (BioFire)	Not detected
*Streptococcus pneumoniae* DNA (BioFire)	Not detected
Cytomegalovirus (CMV) DNA (BioFire)	Not detected
Enterovirus RNA (BioFire)	Not detected
Herpes simplex virus 1 DNA (BioFire)	Not detected
Herpes simplex virus 2 DNA (BioFire)	Not detected
Human herpesvirus 6 DNA (BioFire)	Not detected
Human parechovirus RNA (BioFire)	Not detected
Varicella zoster virus DNA (BioFire)	Not detected
*Cryptococcus neoformans/gattii *(BioFire)	Not detected
Herpes simplex type 1 DNA (PCR)	Not detected
Herpes simplex type 2 DNA (PCR)	Not detected
Varicella-zoster DNA (PCR)	Not detected
Enterovirus RNA (RT-PCR)	Not detected
Parechovirus RNA (RT-PCR)	Not detected

The initial MRI performed through the TIA pathway had shown no acute abnormality, and no medial temporal lobe abnormality was reported at that stage. Approximately six months later, repeat contrast-enhanced MRI was performed during the admission for suspected AIE. This demonstrated bilateral medial temporal lobe enlargement with fluid-attenuated inversion recovery (FLAIR) hyperintensity involving the amygdalae and hippocampi, consistent with limbic encephalitis (Figure 5). The full sequence list for the initial MRI was not available in the clinical record, but the later medial temporal lobe abnormalities were clearly demonstrated on repeat imaging. 

**Figure 5:**
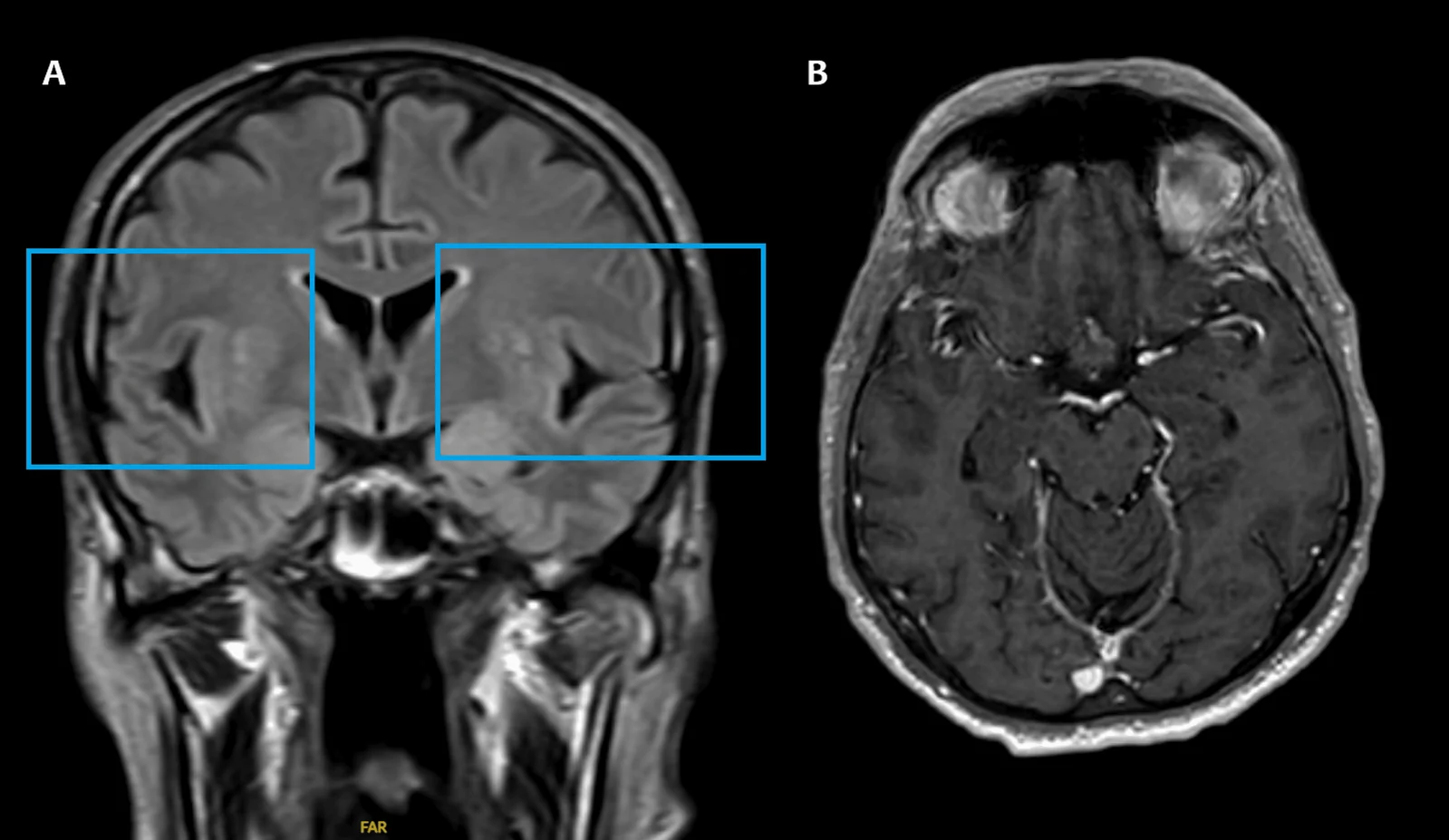
MRI brain confirming the encephalitis Brain MRI demonstrating features of autoimmune limbic encephalitis. Coronal FLAIR sequence (A) shows bilateral hyperintense signal abnormalities involving the mesial temporal lobes and hippocampi (marked in blue). Axial post-contrast T1-weighted image (B) demonstrates no significant abnormal parenchymal enhancement. The imaging findings are consistent with limbic encephalitis in the setting of LGI1 antibody-associated autoimmune encephalitis. MRI: magnetic resonance imaging; FLAIR: fluid-attenuated inversion recovery

A comprehensive AIE antibody panel was performed. Serum AIE antibody testing, performed at Sheffield Immunology, detected anti-LGI1 antibodies. The remaining serum antibodies tested were negative (Table 4).

**Table 4:** Serum autoimmune encephalitis screen AMPA: anti-α-amino-3-hydroxy-5-methyl-4-isoxazolepropionic acid receptor; LG1L antibodies: leucine-rich glioma-inactivated 1 antibodies; GABA-B 1/2: gamma-aminobutyric acid type B receptor subunit 1/2; CASPR2: contactin-associated protein-like 2

Serum encephalitis antibody test	Result
Serum AMPA1/2 receptor antibody	Negative
Serum Anti-DPPX antibody	Negative
Serum LGI1 antibody	Positive
Serum NMDA receptor antibody	Negative
Serum CASPR2 antibody	Negative
Serum GABAB1/2 receptor antibody	Negative

Based on the clinical presentation, characteristic seizure semiology, refractory aphasic episodes, hyponatraemia, EEG findings, bilateral mesial temporal MRI changes, and positive LGI1 antibodies, a diagnosis of LGI1 antibody-associated autoimmune limbic encephalitis was established (Table 5).

**Table 5:** Timeline for the patient A brief timeline for the patient's symptoms and actions taken.

Clinic symptom	Action taken	Outcome
Aphasic episode	Referred to TIA clinic	Started on stroke medication
Seizures	Referred to seizure clinic	Started on antiepileptic medication
Abnormal bloods	Work-up for hyponatraemia	Antiepileptic medication as culprit; medication changed
Ongoing seizure	Extensive workup confirmed autoimmune encephalitis	Steroids started; improvement noted

After infection had been excluded, the patient was treated with intravenous methylprednisolone 1000 mg daily for five days, followed by oral prednisolone 60 mg once daily. He initially responded well, with a significant reduction in seizure frequency to approximately two to four episodes daily, alongside normalisation of serum sodium. However, he subsequently deteriorated again, experiencing approximately 18-25 episodes daily with increasing blank spells. In view of this relapse and ongoing disease activity, intravenous immunoglobulin was prescribed at a total dose of 2 g/kg over five days.

He was reassessed in the neurology clinic, where he continued to report persistent seizure episodes, occurring approximately 2 to 10 times daily. These included two episodes involving the legs, resulting in falls. A typical FBDS was witnessed during the clinic appointment, causing him to fall backwards in the chair, followed by rapid recovery. Given the persistence of symptoms despite corticosteroids and intravenous immunoglobulin, he was referred to the Oxford Autoimmune Neurology Clinic at the John Radcliffe Hospital for further assessment.

Second-line immunotherapy with rituximab was subsequently commenced, given as 500 mg in two infusions two weeks apart. This was followed by a planned prednisolone taper, reducing by 5 mg every two weeks until 20 mg once daily, then by 5 mg monthly until 10 mg once daily, followed by a reduction of 1 mg every two weeks until cessation. Continued clinical improvement was observed, with a progressive decrease in seizure burden and no further generalised tonic-clonic seizures reported.

## Discussion

LGI1 antibody-associated autoimmune limbic encephalitis is a well-recognised and increasingly identified subtype of AIE, typically characterised by a subacute onset of seizures and progressive cognitive impairment [1]. Although FBDS are considered the classical seizure phenotype, growing evidence indicates that the clinical spectrum of LGI1 encephalitis extends far beyond this presentation [14]. Patients may exhibit a wide range of neuropsychiatric and sleep-related manifestations, including memory impairment, confusion, hallucinations, and sleep disturbances such as rapid eye movement sleep behaviour disorder, hypersomnolence, and insomnia, as well as less common movement disorders such as persistent focal dystonia [14].

In this context, our case highlights an atypical and diagnostically challenging presentation, where the first documented manifestation was isolated recurrent speech disturbance. The episodes were best characterised as expressive aphasic seizures or speech arrest, as awareness was preserved and comprehension appeared intact from the patient’s and wife’s account; however, formal receptive language testing was not performed during the brief attacks. Aphasic seizures are an uncommon form of focal seizure resulting from transient dysfunction of the language-dominant cortex, typically arising from the dominant temporal lobe. These may present as speech arrest, expressive or receptive aphasia, or mixed language impairment [15]. Recognition of such non-motor seizure semiology is essential, as recurrent aphasic seizures may represent an early manifestation of LGI1 encephalitis.

However, transient neurological symptoms lasting less than 24 hours are not synonymous with TIA, and epileptic seizures must remain an important differential diagnosis [16]. The recurrent, stereotyped nature of the episodes, their brief duration, and increasing frequency strongly favoured an epileptic aetiology rather than cerebral ischaemia. This aligns with existing literature indicating that seizures are among the most common presenting features of LGI1 encephalitis [17]. While seizures are often motor in nature, they may also present with non-motor or autonomic features, including rising epigastric sensations and sinus tachycardia, reflecting involvement of limbic networks [18]. This broad clinical spectrum underscores the need for heightened clinical suspicion, particularly in patients presenting with atypical or non-motor seizure manifestations.

The subsequent development of hyponatraemia provided an additional diagnostic clue. Hyponatraemia is one of the most frequently reported laboratory abnormalities in LGI1 encephalitis, occurring in approximately 60% of cases [3,4]. Although nonspecific, its presence in conjunction with unexplained neurological symptoms, particularly focal seizures, behavioural change, or cognitive decline, should prompt consideration of an autoimmune aetiology. In this case, the biochemical profile supported SIADH rather than excluding it. Alternative causes of hypotonic hyponatraemia, including hypothyroidism, adrenal insufficiency, renal impairment, gastrointestinal losses, and diuretic use, should be considered. The patient was clinically euvolemic with normal thyroid and adrenal function and preserved renal function, supporting SIADH as the most likely mechanism [11]. 

EEG findings in LGI1 encephalitis are variable but most commonly involve abnormalities in the temporal regions, including interictal epileptiform discharges, focal slowing, and rhythmic ictal activity, consistent with the findings observed in our patient [19]. Neuroimaging further illustrated an important diagnostic consideration: initial CT and MRI studies were unremarkable, whereas subsequent contrast-enhanced MRI revealed bilateral medial temporal lobe enlargement with FLAIR hyperintensity involving the hippocampi and amygdalae, features characteristic of limbic encephalitis. The interval of approximately six months between the initial unrevealing MRI and the later diagnostic MRI highlights the evolving nature of the disease and the value of repeat imaging when clinical suspicion remains high. Although imaging may be normal in some patients, when present, abnormalities most frequently involve the medial temporal lobes and, less commonly, the hypothalamus on T2/FLAIR-weighted sequences [5,20].

The diagnostic reasoning was supported by published criteria for AIE. Taken together, the subacute evolution of seizures with cognitive symptoms, MRI features of limbic encephalitis, exclusion of infectious mimics, and detection of a neuronal surface antibody supported the diagnosis within the framework proposed by Graus et al. [21]. The phenotype was also consistent with the LGI1-antibody-associated syndrome described by van Sonderen et al. [5], including recurrent focal seizures, FBDS, SIADH-related hyponatraemia, medial temporal lobe MRI changes, and serum anti-LGI1 antibody positivity. 

The differential diagnosis remained broad during the patient’s early presentations. A cerebrovascular event was initially considered because of isolated transient aphasia and vascular risk factors; however, the recurrent, brief, stereotyped nature of the episodes and subsequent seizure evolution made this less likely as a unifying diagnosis. Structural, infective, paraneoplastic, and alternative autoimmune causes were also considered. The absence of a structural lesion or malignancy on imaging, non-inflammatory and microbiologically negative CSF, including negative herpes simplex virus testing, and negative results for other AIE antibodies supported LGI1-antibody AIE as the most likely diagnosis.

The patient initially demonstrated a significant clinical and biochemical response following treatment with high-dose intravenous methylprednisolone, followed by oral corticosteroids, with reduction in seizure burden and normalisation of serum sodium. However, the subsequent recurrence of frequent aphasic and faciobrachial dystonic episodes suggested ongoing immune-mediated disease activity rather than isolated electrolyte-driven seizures or medication-related events. This prompted escalation of immunotherapy with intravenous immunoglobulin and later rituximab following specialist autoimmune neurology review. Plasma exchange was not documented as having been used during the reported period. Immunotherapy remains the cornerstone of treatment in LGI1 encephalitis and may include corticosteroids, intravenous immunoglobulin, plasma exchange, or rituximab [22]. In this case, the relapse after initial corticosteroid response highlighted the importance of close follow-up, reassessment of seizure burden, and timely escalation when disease activity persists.

Early initiation of immunotherapy has been shown to improve seizure control, reverse cognitive impairment, and optimise long-term outcomes. Conversely, delays in diagnosis and treatment may result in persistent cognitive deficits and prolonged functional impairment despite subsequent therapy [23].

## Conclusions

Isolated recurrence of aphasic seizures in this patient remains uncommon but might be largely underdiagnosed because transient speech arrests typically occur in cerebrovascular events such as stroke. To our knowledge, this case suggests that recurrent aphasic seizures may be the first presentation of LGI1 antibody-associated autoimmune limbic encephalitis, with the diagnosis supported by high-frequency stereotyped speech arrests, worsening with antiseizure treatments, hyponatraemia, slowly progressing mesial temporal MRI changes, and later positive LGI1 antibodies. LGI1 antibody-associated autoimmune limbic encephalitis should thus be strongly suspected in adults with unexplained recurrent aphasic spells, especially when associated with seizures, cognitive deficits, or hyponatraemia. Prompt recognition and therapy remain important for neurological outcomes and prognosis, with reduced long-term morbidity.
